# Lobular capillary hemangioma with halo phenomenon

**DOI:** 10.1016/j.jdcr.2025.01.005

**Published:** 2025-01-31

**Authors:** Philip R. Cohen, Nikolas B. Gutierrez, Christof P. Erickson, Antoanella Calame

**Affiliations:** a Department of Dermatology, University of California, Davis Medical Center, Sacramento, California; b Touro University California College of Osteopathic Medicine, Vallejo, California; c Maples Center for Forensic Medicine, University of Florida College of Medicine, Gainesville, Florida; d Family Medicine Residency, Naval Hospital, Camp Pendleton, California; e Compass Dermatopathology, San Diego, California

**Keywords:** angiokeratoma, angioma, antigen, capillary, eosin, Fontana, Fontana-Masson, granuloma, halo, hemangioma, hematoxylin, hypopigmentation, lobular, MART-1, Masson, Melan-A, melanin, melanocyte, nevus, phenomenon, pyogenic, recognized, stain, T cell

*To the Editor:* We read with interest the report of halo angiokeratoma.^1^ In addition to vascular lesions, halo phenomenon has been observed in association with epithelial neoplasms, genodermatoses, and melanocytic tumors (Table I).^1^, ^2^, ^3^, ^4^, ^5^ Although the angiokeratoma reported by AlJasser showed a complete absence of melanocytes in the epidermis (Melan-A stain),^1^ the author failed to acknowledge the possibility of vascular lesion-related halo phenomenon being caused by postinflammatory hypopigmentation.^5^

**Table I:** Lesions associated with halo phenomenon

Epithelial neoplasms: benign
Seborrheic keratosis
Epithelial neoplasm: malignant
Basal cell carcinoma
Melanocytic neoplasms: benign nevus
Coronavirus disease 2019 (infection or vaccine)-related
Halo
Paraneoplastic-related
Therapy (drug)-related
Melanocytic neoplasms: malignant melanoma
Metastatic
Primary
Scar (from excision)-related
Syndrome associated
Neurofibromatosis (café-au-lait macules)
Turner syndrome (halo nevi)
Vascular lesions
Angiokeratoma
Angioma
Capillary malformation-arteriovenous malformation
Congenital hemangioma
Infantile hemangioma
Lobular capillary hemangioma

We previously reported a woman whose lobular capillary hemangioma with halo phenomenon that was caused by postinflammatory hypopigmentation; the lesion demonstrated not only the preservation of melanocytes but also the loss of melanin pigment expression (Fig 1).^5^ In contrast to the patient with halo angiokeratoma who had elimination of all epidermal melanocytes, the woman with halo lobular capillary hemangioma had numerous epidermal melanocytes.^5^

**Fig 1:**
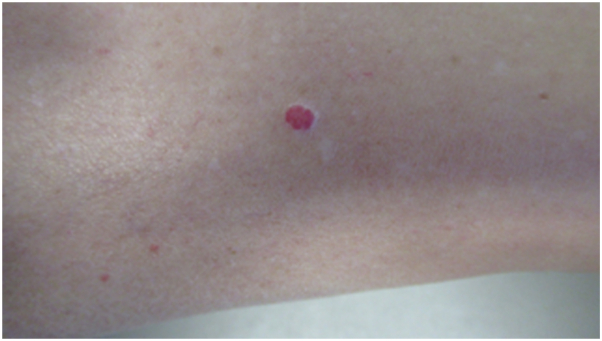
Clinical appearance of halo phenomenon in a lobular capillary hemangioma. A 50-year-old Caucasian woman had an acquired asymptomatic red lesion on her distal right leg of unknown duration that would occasionally become irritated; previously, it had twice spontaneously bled. Examination of the medial aspect of her distal lower leg (with the calf towards the left and the ankle towards the right) showed a 5.0 × 4.0 millimeter red nodule; it was surrounded by a white epithelial collarette and an asymmetric hypopigmented patch. A tangential excision, using the shave technique, completely removed the lesion. The details of this report have been described,^2^ yet the photograph has not been previously published.

Microscopic examination of the basal layer of the epidermis of the halo lobular capillary hemangioma revealed melanin expression in the center of the lesion and the skin peripheral to the areas of hypopigmentation (Fig 2 and 3); there was an absence of melanin expression in the basal layer of the epidermal collarettes and the immediately bilateral adjacent epidermis (Fontana-Masson stain). Importantly, in contrast to the benign halo angiokeratoma, the halo lobular capillary hemangioma demonstrated postinflammatory hypopigmentation: melanocytes were present not only within the entire vascular lesion but also in the surrounding area of hypopigmentation containing the epidermal collarette (melanoma antigen recognized by T cells 1 stain).^5^

**Fig 2:**
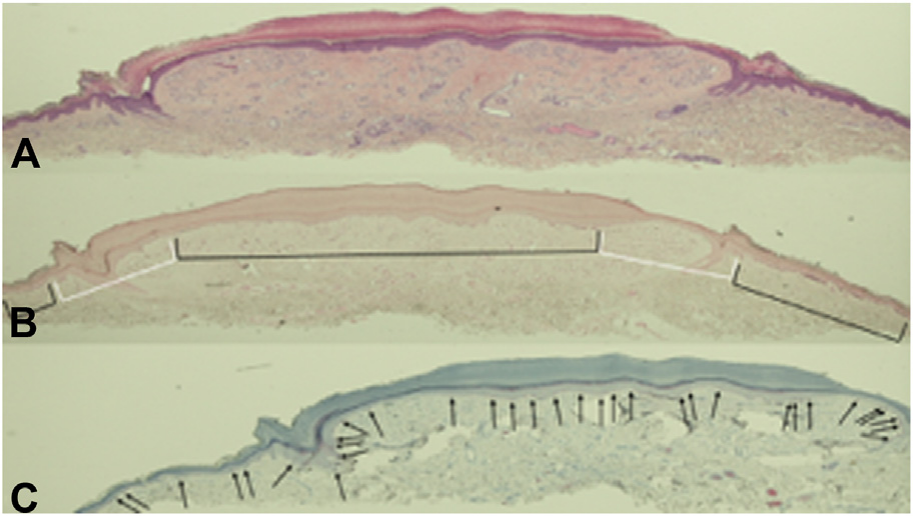
Microscopic findings of lobular capillary hemangioma with halo phenomenon. Hematoxylin and eosin staining (**A**) show a proliferation of capillaries in a fibrotic dermal stroma; there is solar elastosis and a minimal inflammatory infiltrate in the dermis. There is compact orthokeratosis overlying an acanthotic epidermis in the central portion of the lesion. A collarette of epithelium surrounds the vascular tumor in the lateral portion of the lesion; mounds of orthokeratosis project from the epidermis (corresponding to the white scaling observed clinically) and elongated rete ridges extend into the dermis separating the lesional dermis from the adjacent dermis. Fontana-Masson staining (**B**) reveals an absence of melanin expression in the basal layer of the epidermal collarettes and the immediately bilateral adjacent epidermis (*white brackets*); however, the basal layer of the epidemis in the center of the lesion and the skin peripheral to the areas of hypopigmentation demonstrates melanin expression (*black brackets*). MART-1 staining (**C**) shows melanocytes present in the basal layer of the epidermis that is both overlying the entire vascular lesion and also in the hypopigmented area that not only surrounds the vascular lesion but also includes the epidermal collarette. The microscopic changes of this report have been described,^2^ yet the photomicrographs have have not been previously published (**A,** hematoxylin and eosin, ×2; **B,** Fontana-Masson, × 2; **C,** MART-1, ×2).

**Fig 3:**
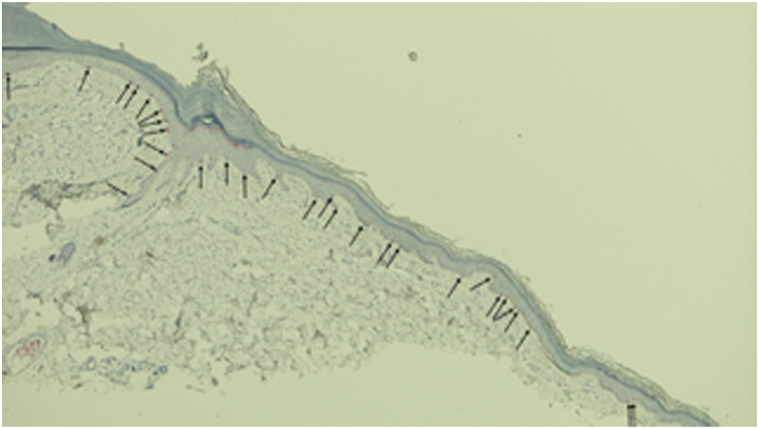
Halo phenomenon in a lobular capillary hemangioma shows preservation of melanocytes. A higher magnification view of the MART-1 staining of a lobular capillary hemangioma with halo phenomenon shows melanocytes (*black arrows*) present along the basal layer of the epidermis. Therefore, the loss of melanin pigment expression with the preservation of melanocytes in the white epidermal collarette and hypopigmented perilesional areas is consistent with postinflammatory hypopigmentation being responsible for the pathogenesis of the halo phenomenon that occurred in the lobular capillary hemangioma. The microscopic changes of this report have been described,^2^ yet the photomicrographs have not been previously published (MART-1, × 20).

In summary, there are two mechanisms of pathogenesis in vascular lesion-associated halo phenomenon: postinflammatory hypopigmentation and complete absence of melanocytes.^1^^,^^5^

## Conflicts of interest

None disclosed.
